# Analysis of synonymous codon usage patterns in mitochondrial genomes of nine *Amanita* species

**DOI:** 10.3389/fmicb.2023.1134228

**Published:** 2023-03-08

**Authors:** Qiang Li, Yingyong Luo, Ajia Sha, Wenqi Xiao, Zhuang Xiong, Xiaodie Chen, Jing He, Lianxin Peng, Liang Zou

**Affiliations:** Key Laboratory of Coarse Cereal Processing, Ministry of Agriculture and Rural Affairs, School of Food and Biological Engineering, Chengdu University, Chengdu, Sichuan, China

**Keywords:** codon usage, mitochondrial genome, evolution, genetics, natural selection

## Abstract

**Introduction:**

Codon basis is a common and complex natural phenomenon observed in many kinds of organisms.

**Methods:**

In the present study, we analyzed the base bias of 12 mitochondrial core protein-coding genes (PCGs) shared by nine *Amanita* species.

**Results:**

The results showed that the codons of all *Amanita* species tended to end in A/T, demonstrating the preference of mitochondrial codons of *Amanita* species for a preference for this codon. In addition, we detected the correlation between codon base composition and the codon adaptation index (CAI), codon bias index (CBI), and frequency of optimal codons (FOP) indices, indicating the influence of base composition on codon bias. The average effective number of codons (ENC) of mitochondrial core PCGs of *Amanita* is 30.81, which is <35, demonstrating the strong codon preference of mitochondrial core PCGs of *Amanita*. The neutrality plot analysis and PR2-Bias plot analysis further demonstrated that natural selection plays an important role in *Amanita* codon bias. In addition, we obtained 5–10 optimal codons (ΔRSCU > 0.08 and RSCU > 1) in nine *Amanita* species, and GCA and AUU were the most widely used optimal codons. Based on the combined mitochondrial sequence and RSCU value, we deduced the genetic relationship between different *Amanita* species and found large variations between them.

**Discussion:**

This study promoted the understanding of synonymous codon usage characteristics and evolution of this important fungal group.

## Introduction

The genetic information contained in biological DNA is transferred to the sequence of 20 amino acids according to the “central rule.” Of the 64 nucleotide triplets (codons) contained in DNA, 61 can encode standard 20 amino acids, while the other three are translation stop signals. Eighteen of the 20 amino acids are coded by multiple synonymous codons, while tryptophan and methionine are coded by only one codon in most species (Quax et al., 2015). The degeneration of the genetic code allows the same amino acid to be encoded by different codons or synonymous codons (Yao et al., 2019; Bailey et al., 2021). However, in different organisms, different genes, or even the same gene from different species, the probability of synonymous codons being used is not equal or random, and some codons are repeatedly preferred to other codons to encode amino acids (Behura and Severson, 2012; Barbhuiya et al., 2020). There is a common phenomenon called codon usage bias (CUB), in which synonymous codons appear with different frequencies (Hershberg and Petrov, 2008; Choudhury et al., 2018). Mutations in the gene coding region, particularly in the second or third nucleotide of the existing codon, can lead to the alteration of one synonymous codon to another; however, this will not have an effect on the amino acid or peptide primary sequence specified by the new and modified codon (Iriarte et al., 2021; Parvathy et al., 2022). In evolution, these synonymous mutations, or “silent mutations” that do not change the initial sequence of proteins or peptides will lead to the variation of synonymous codons in organisms. Thus, codon usage bias occurs as a result of biased mutation patterns; as some codons may be more prone to mutation than others, selection can maintain such bias (Behura and Severson, 2013; Arella et al., 2021). A codon use bias can also be derived from GC heterogeneity and GC-biased gene transformation (gBGC) based on local recombination rates (Akashi, 1997; Dilucca et al., 2021; Parvathy et al., 2022). As a result, synonymous codons evolved through a balance of mutations, natural selection, and genetic drift in gene translation efficiency, which may contribute significantly to genome evolution (Krasovec and Filatov, 2019; Dilucca et al., 2020; Gomez et al., 2020). The mutation mechanism of codon bias assumes that codon bias is caused by nucleotide bias or the rate or repair of point mutation and explains the interspecific variation in codon use (Palidwor et al., 2010; Rao et al., 2011; Wang et al., 2011). The theory of natural selection also assumes that synonymous mutations, which affect the adaptability of organisms, will be promoted or suppressed throughout the evolution process when the force affecting biological adaptability is large enough, thus resulting in variations in the use of codons in genomes or genes (Lal et al., 2016; Xu et al., 2021).

Codon bias is thought to affect a wide range of cellular processes, including mRNA stability, transcription, translation efficiency and accuracy, as well as protein expression, structure, function, and cotranslation folding (Chu and Wei, 2021; Franzo et al., 2021; Hia and Takeuchi, 2021). By affecting chromatin structure and mRNA folding, codon bias can influence transcription levels and translation efficiency by regulating the elongation rate of translation (Uddin et al., 2015). Thus, codon bias is caused by genome adaptations to transcription and translation mechanisms or only adaptations (Biswas et al., 2019). Selecting gene sequences without changing amino acids has far-reaching significance for the study of the molecular evolution of genes. As closely related organisms use codons in similar ways, codon bias analysis can reveal horizontal gene transfer and evolutionary relationships between organisms (LaBella et al., 2019; Kokate et al., 2021). Most highly expressed proteins are encoded by genes with appropriate codons. Therefore, heterologous genes can be optimized in genetic engineering or recombinant DNA technology to enhance the protein expression of heterologous genes. With the rapid development of high-throughput sequencing technology, fully analyzing codon bias is helpful to understanding the genetic mechanism of species evolution, environmental adaptation, genetics, and so on (Huo et al., 2021; Hugaboom et al., 2023). However, so far, the genetic characteristics of codon bias in fungal species, especially in large higher fungi, have not been fully understood (Araujo et al., 2021; Wint et al., 2022).

*Amanita* is a population rich in species diversity, and approximately 600 species are described in the genus of *Amanita* (Wolfe et al., 2012). Some *Amanita* species are considered edible, while some species are considered toxic (Cai et al., 2016; Ye and Liu, 2018). Most species of *Amanita* are ectomycorrhizal fungi, while a few are saprophytic, and they all evolved from their symbiotic ancestors (Wolfe et al., 2012; De Mares et al., 2015). *Amanita* has been used as a model organism to study the origin, evolution, and genetics of fungal life patterns (Hess et al., 2014, 2018). During the evolution of *Amanita*, a series of genes have evolved to better adapt to its diverse lifestyles (Kohler et al., 2015). The mitochondrial genome is considered the “second genome” of fungi besides the nuclear genome, encoding a series of oxidative phosphorylation-related genes for fungal energy metabolism (Sankoff et al., 1992; Boore, 1999; Poliseno et al., 2017; Qian et al., 2018). It has been reported that most fungi contain 15 core protein-coding genes (CDSs), including *atp6*, *atp8*, *atp9*, *cob*, *cox1*, *cox2*, *cox3*, *nad1*, *nad2*, *nad3*, *nad4*, *nad4L*, *nad5*, *nad6*, and *rps3* (Li et al., 2018, 2019). Mutations in fungal mitochondrial core protein-coding genes may lead to the destruction of fungal cell homeostasis and induce cell damage, growth inhibition, and even fungal death (Osiewacz, 2002; Chatre and Ricchetti, 2014). Therefore, the study of base bias of fungal mitochondrial core protein-coding genes will help to understand the genetic and evolutionary characteristics of fungal mitochondrial genes, especially in this diverse *Amanita* population.

In this study, we systematically analyzed and compared the codon usage characteristics of mitochondrial core protein-coding genes of nine *Amanita* species, including *A. basii*, *A. bisporigera*, *A. brunnescens*, *A. muscaria*, *A. phalloides*, *A. pseudoporphyria*, *A. sinensis*, *A. inopinata*, and *A. thiersii*. The phylogenetic tree of *Amanita* species was constructed based on the relative synonymous codon usage of mitochondrial core genes of *Amanita* species and was compared with the phylogenetic tree constructed based on the core protein encoding gene sequences. The results promoted the understanding of the codon inheritance, evolution, and environmental adaptation of important energy metabolism genes of diverse fungal groups.

## Materials and methods

### Sequence processing

To date, nine complete mitochondrial genomes from the *Amanita* genus have been deposited in the National Center for Biotechnology Information (NCBI) database, including eight species previously reported by us and one reported by other scholars (Li et al., 2020). We downloaded the core protein-coding genes of the eight *Amanita* mitochondrial genomes from the NCBI database under the accession numbers NC_045194–NC_045201 and NC_058596. The mitochondrial coding genes obtained were further screened, and those with sequences less than 100 coding amino acids were excluded from downstream analysis (Bu et al., 2021). Finally, we obtained 12 core protein-coding genes in each mitochondrial genome of *Amanita* for downstream analysis: *atp6*, *cob*, *cox1*, *cox2*, *cox3*, *nad1*, *nad2*, *nad3*, *nad4*, *nad5*, *nad6*, and *rps3*.

### Codon usage indices

The GC3s parameter determines the proportion of codons with guanine and cytosine at third synonymous positions, excluding Met, Trp, and termination codons (Huo et al., 2021). The codon adaptation index (CAI) is commonly used to assess bias toward codons, which are known to be preferred in highly expressed genes. It ranges from 0 to 1.0; the larger the value is, the more frequently the synonymous codon is used. The codon bias index (CBI) is used as a standard to evaluate gene expression, which reflects the components of a gene with high expression of superior codons. The frequency of optimal codons (FOP) is calculated by counting the ratio of the optimal codon number to the total synonymous codon number in the gene. The effective number of codons (ENC) refers to the number of effective codons used in a gene and ranges from 20 to 61. An ENC value equal to 20 means that only one codon is used for each amino acid, and 61 means that each codon is used on average. If the value of ENC is lower than 35, then the codon usage preference is strong; if it is higher than 35, then the codon usage preference is weak. By dividing the amino acids encoded by the same codons and their probability of appearing in the same codons, the relative synonymous codon usage (RSCU) value was calculated. An RSCU value greater than 1 indicates a positive codon bias, an RSCU value less than 1 indicates a negative codon bias, and an RSCU value equal to 1 indicates a random codon usage. We calculated general average hydropathicity (GRAVY) values by summing the hydropathy values of all of the amino acids in the polymerase gene sequences and multiplying them by the number of residues in the gene sequences. GRAVY values range from −2 to 2; positive and negative values represent hydrophobic and hydrophilic proteins, respectively. The aromaticity (AROMO) value indicates the frequency of aromatic amino acids (Phe, Tyr, and Trp). GRAVY and AROMO values are also indicators of amino acid usage, and changes in amino acid composition will also affect the results of codon usage analysis. All of the codon usage indicators above were calculated through CodonW1.4.2 (Peden, 1999) and CAIcal server (Puigbo et al., 2008).

### Neutrality plot analysis

The neutrality plot (GC12 vs. GC3) can illustrate the balance between mutation and selection when codon bias is formed. GC12 represents the average GC content in the first and second positions of the codon (GC1 and GC2), while GC3 represents the GC content in the third position. If we find a strong statistical correlation between GC12 and GC3, then the main driving force of the species is mutation pressure. In contrast, if there is no correlation between GC12 and GC3, then the main driving force of the tested species is natural selection.

### ENC-GC3s plot analysis

The ENC-GC3s plot (ENC vs. GC3s) is usually used to check whether the codon usage of a specific gene is affected only by mutation or other factors, such as natural selection. The ENC-GC3s diagram consists of the ordinate ENC value and abscissa GC3s value. Furthermore, we calculated the expected curve on the ENC-GC3s diagram based on the following formula. If we find that the corresponding points are distributed around the expected curve, we can conclude that the mutation pressure is an independent force in the formation of codon bias. If the corresponding point is lower or far from the expected curve, there should be some other factors, such as natural selection, playing a key role in the formation of codon bias.


$$ENC_{\exp}=2+GC3_{S}+\frac{29}{GC3+S2{\left(1-GC3_{S}\right)}^{2}}$$

We further tested the variations between the expected value and the actual value of ENC, which is reflected by the ENC_Ratio_ index. The ENC_Ratio_ value reflects the variation range between the expected value and the actual value of ENC.


$$ENC_{Ratio}=\frac{ENC_{\exp}-ENC_{obs}}{ENC}$$

### PR2-bias plot analysis

We further performed the Parity Rule 2 bias (PR2-Bias) plot analysis based on [A3/(A3 + U3) vs. G3/(G3 + C3)]. The value when the center point in the plot is A = T and C = G, that is, the codon has no usage bias, and the other vectors emitted from the center point indicate the degree and direction of the gene bias.

### Correspondence analysis

Correspondence analysis (COA) is a widely accepted multivariate statistical analysis method to determine codon usage patterns. As these genes have 59 sense codons (Met and Trp are excluded from the 61 total codons), we put all genes in the 59-dimensional hyperspace in the figure. This method can explore the main trend of codon usage change in the core CDS of *Amanita* and distribute codons along the axis according to the RSCU value.

### Determination of optimal codons

We arranged all of the tested genes in order from large to small according to the CAI value, and 10% of the genes were selected from the front and rear ends to establish a high-and low-expression gene dataset. We further calculated the D-value between the RSCU of the two datasets (ΔRSCU), where ΔRSCU values greater than 0.08 were defined as codons with high expression. Codons with RSCU values greater than 1 are regarded as high-frequency codons. A codon with ΔRSCU>0.08 and RSCU>1 was defined as the optimal codon.

### Phylogenetic analysis

We compared the phylogenetic relationships of *Amanita* species between codon usage-based and mitochondrial sequence-based methods. Based on the RSCU values of the nine *Amanita* species, we used SPSS v19.0 software based on the hierarchical clustering method to draw the relationship tree between different species. We then used the combined mitochondrial gene datasets to construct phylogenetic trees of the 9 *Amanita* species according to the method described by our previous studies (Li et al., 2022a,b). Briefly, individual mitochondrial genes were first aligned using MAFFT v7.037(Katoh et al., 2019), and then the aligned mitochondrial sequences were concatenated into a combined mitochondrial gene set based on Sequence Matrix v1.7.8 (Vaidya et al., 2011). Potential phylogenetic conflicts between different mitochondrial genes were detected by a preliminary partition homogeneity test. We then used Partition Finder 2.1.1 (Lanfear et al., 2017) to detect the best-fit model of partitioning schemes and evolution for the combined mitochondrial gene set. We constructed the phylogenetic tree by the Bayesian inference (BI) method using MrBayes v3.2.6 (Ronquist et al., 2012). Specifically, two independent runs with four chains (three heated and one cold) each were conducted simultaneously for 2 × 10^6^ generations. Each run was sampled every 100 generations. We assumed that stationarity had been reached when the estimated sample size was greater than 100 and the potential scale reduction factor approached 1.0. The first 25% of samples were discarded as burn-in, and the remaining trees were used to calculate Bayesian posterior probabilities (BPP) in a 50% majority-rule consensus tree.

## Results

### Nucleotide composition of *Amanita* core PCGs

We selected 12 mitochondrial core PCGs from the nine *Amanita* species for codon usage analysis. Statistical analysis found that the average length of these core PCGs ranged from 376 bp to 2004 bp, with the *nad3* gene having the shortest average length and the *nad5* gene having the longest average length. Among the 12 core PCGs, eight genes showed sequence length variations among different *Amanita* species, while four genes had the same gene length among nine *Amanita* species. Among the 12 core PCGs, the *rps3* gene showed the largest length variation among different species, and different *Amanita* species have different gene lengths from other species. We further calculated the base composition of the 12 core PCGs of nine *Amanita* species and found that the mitochondrial core PCGs of nine *Amanita* species were rich in the T base, with an average content of 41.08%, followed by the A base, with an average content of 31.73%. The G and C base contents of mitochondrial core PCGs in *Amanita* species were relatively small, with an average of 13.90 and 13.29%, respectively. The average GC content of the 12 core PCGs ranged from 18.67 to 33.47%, with the rps3 gene containing the lowest GC content and the cox1 gene containing the highest GC content.

### Codon usage index analysis

**Figure 1:**
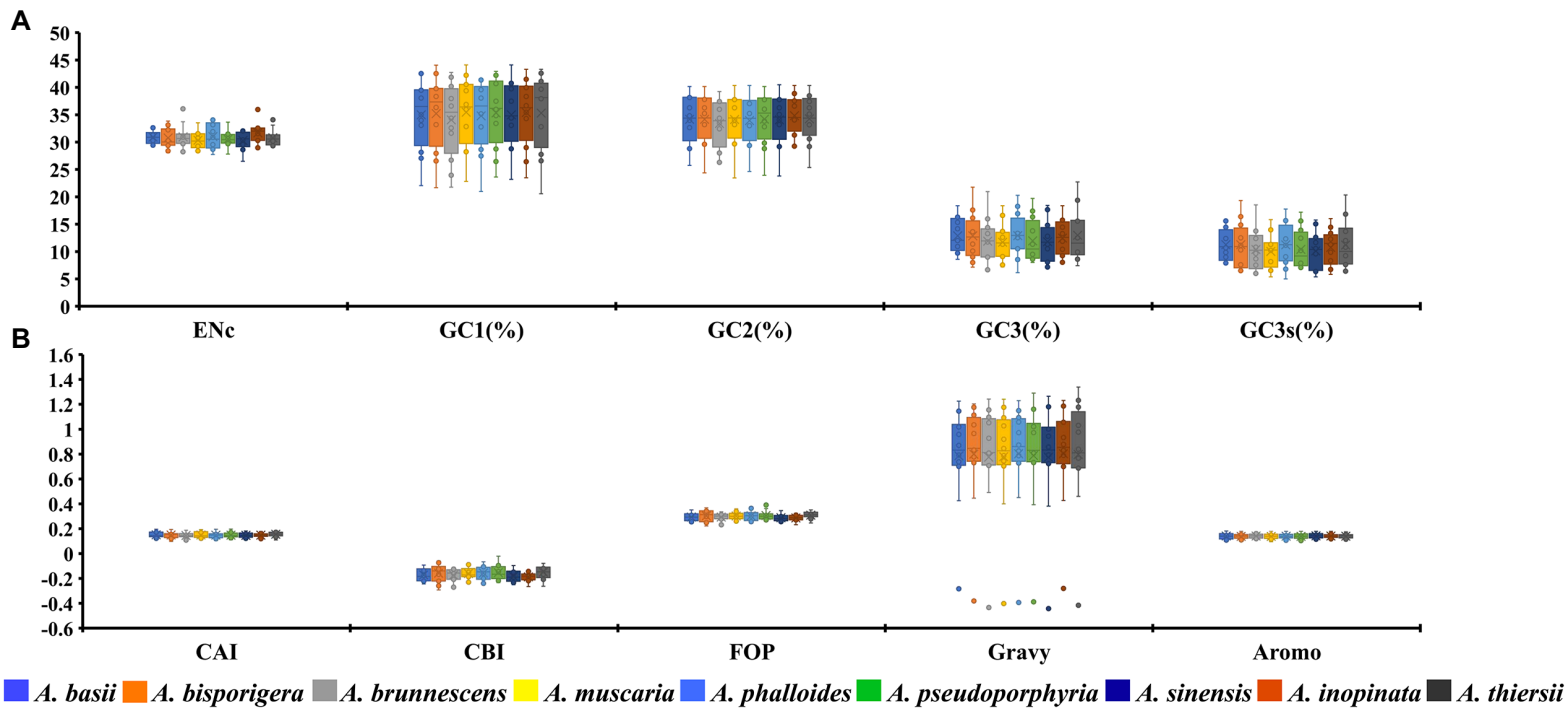
Box plot of codon usage indicators of 12 mitochondrial core protein-coding genes in different species of *Amanita*. A, ENc, GC1, GC2, GC3, and GC3s indicators; B, CAI, CBI, FOP, Gravy, and Aromo indicators.

The GC1, GC2, and GC3 contents of 12 core PCGs in 9 *Amanita* species were 35.06, 34.13 and 12.38%, respectively (Figure 1A). The average GC3s value of 12 PCGs in nine *Amanita* species was 10.73%, which showed that the codon of the mitochondrial core PCGs of *Amanita* tended to end with an A or T base. We further calculated the indices of A3s, T3s, G3s, and C3s of 12 core PCGs of *Amanita* species and found that the mitochondrial codons of *Amanita* were more inclined to end with A, followed by T, C and G, with values of 55.84, 52.31, 8.11, and 3.45%, respectively. We calculated the CAI values of 12 core PCGs of nine *Amanita* species and found that the CAI values of core PCGs ranged from 0.11 to 0.19. Among them, *nad2* had the lowest CAI value, while the *cox2* gene had the highest CAI value, indicating that the *nad2* gene had high codon bias. In terms of species, the core PCGs of *A. thiersii* have the highest CAI value, followed by those of *A. basii*, while the CAI values of *A. brunnescens* and *A. sinensis* are the lowest, indicating that *A. brunnescens* and *A. sinensis* have strong codon bias of mitochondrial core PCGs (Figure 1B). The CBI values of the 12 core PCGs we detected ranged from-0.24 to-0.1. Among them, *nad2* had the lowest CBI value, and *cox1* had the highest CBI value. Regarding species, the CBI values of nine *Amanita* species ranged from −0.19 to −0.15, among which *A. inopinata* had the lowest CBI value and *A. thiersii* had the highest CBI value. In terms of PCGs, the average FOP values ranged from 0.26 to 0.35 among the 12 core PCGs detected, while *nad2* had the lowest FOP value and *cox1* had the highest FOP value. Among the nine *Amanita* species, *A. inopinata* had the lowest FOP value, while *A. thiersii* had the largest FOP value, which were 0.29 and 0.31, respectively. We further calculated the GRAVY values of 12 core PCGs in nine *Amanita* species. The results showed that 11 of the 12 core PCGs had positive GRAVY values, indicating that they might be hydrophobic proteins, while only the *rps3* gene was considered hydrophilic. The AROMO value range of different PCGs is 0.11–0.18; the *nad2* gene has the highest AROMO value, and the *cox3* gene has the lowest AROMO. The AROMO values of different *Amanita* species varied little, with an average of 0.14.

### Correlation analysis of codon usage indices

**Figure 2:**
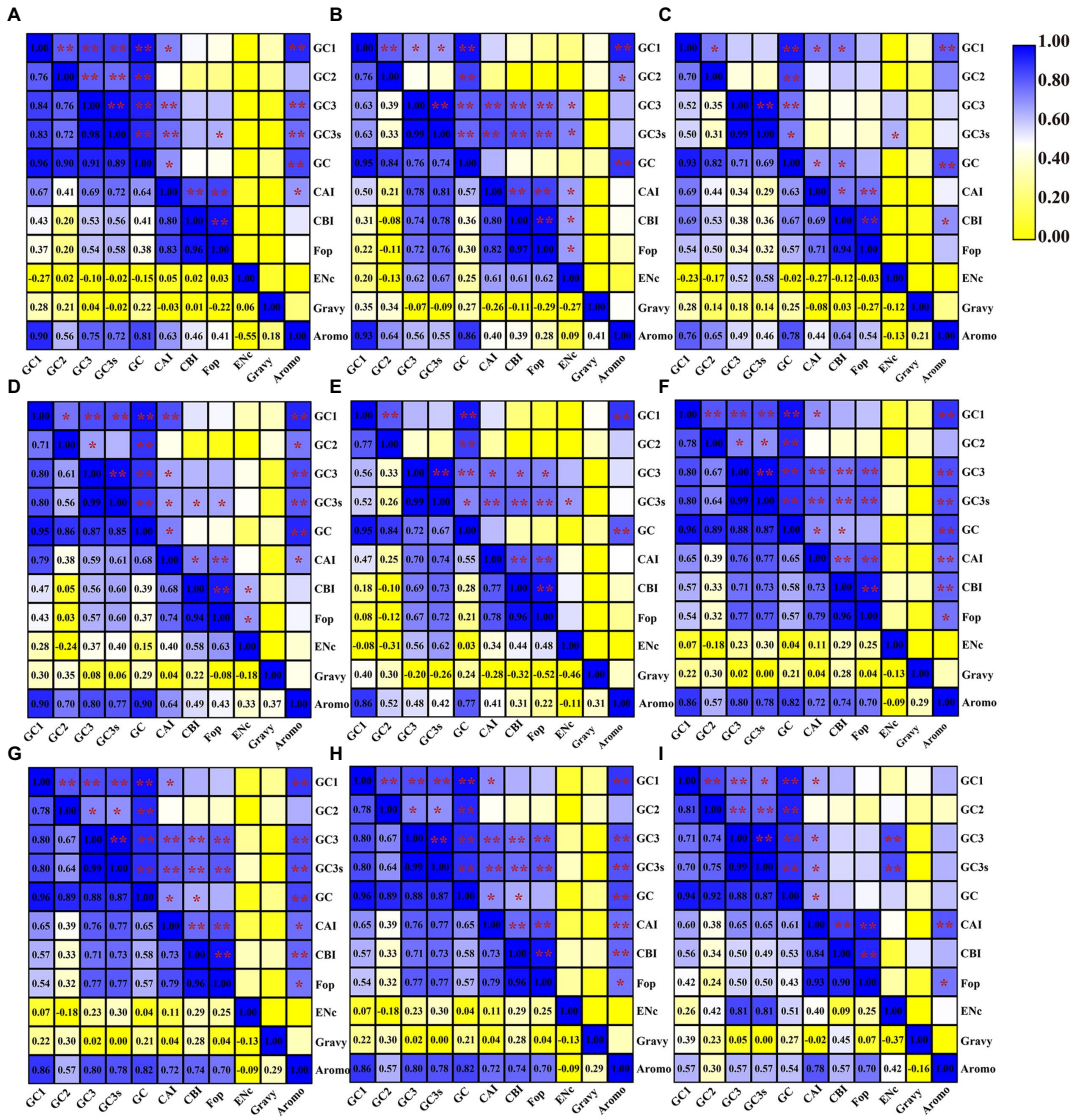
Pearson’s correlation analysis heatmap of different codon usage indicators of nine *Amanita* species. The color of the color block changes from yellow to blue, indicating that the correlation index is increasing. One asterisk indicates a significant correlation between the two indicators at the *p* < 0.05 level, while two asterisks indicate a significant correlation between the two indicators at the *p* < 0.01 level. **(A)**, *A. basii*; **(B)**, *A. bisporigera*; **(C)**, *A. brunnescens*; **(D)**, *A. muscaria*; **(E)**, *A. phalloides*; **(F)**, *A. pseudoporphyria*; **(G)**, *A. sinensis*; **(H)**, *A. inopinata*; and **(I)**, *A. thiersii*.

We found that the content of GC1 in mitochondrial codons was significantly correlated with the GC2, GC3, GC3s and GC content in seven of nine *Amanita* species: *A. basii*, *A. bisporigera*, *A. muscaria*, *A. pseudoporphyria*, *A. sinensis*, *A. inopinata*, and *A. thiersii* (Figure 2). In *A. brunnescens* and *A. phalloides*, we did not observe a significant correlation between GC1 and GC2 or GC3 (Figures 2C,E). In addition, the content of GC1 had a significant correlation with the CAI index of the seven *Amanita* species, *A. basii*, *A. brunnescens*, *A. muscaria*, *A. pseudoporphyria*, *A. sinensis*, *A. inopinata*, and *A. thiersii*, which showed that the GC1 content could significantly affect the codon basis in *Amanita* mitochondrial PCGs. There was a significant correlation between the GC2 and GC contents of all *Amanita* species (*p* < 0.01), indicating that the GC2 content could affect the GC composition of species to some extent. In all nine *Amanita* species tested, the GC3 content in the mitochondrial codon was considered to be significantly correlated with the GC3s and GC contents (*p* < 0.01). In all *Amanita* species, the CAI index of mitochondrial codons was significantly correlated with the CBI index and FOP index (*p* < 0.05). The CBI index was found to have a significant correlation with the FOP index in 9 *Amanita* species (*p* < 0.05).

### Neutrality plot analysis

**Figure 3:**
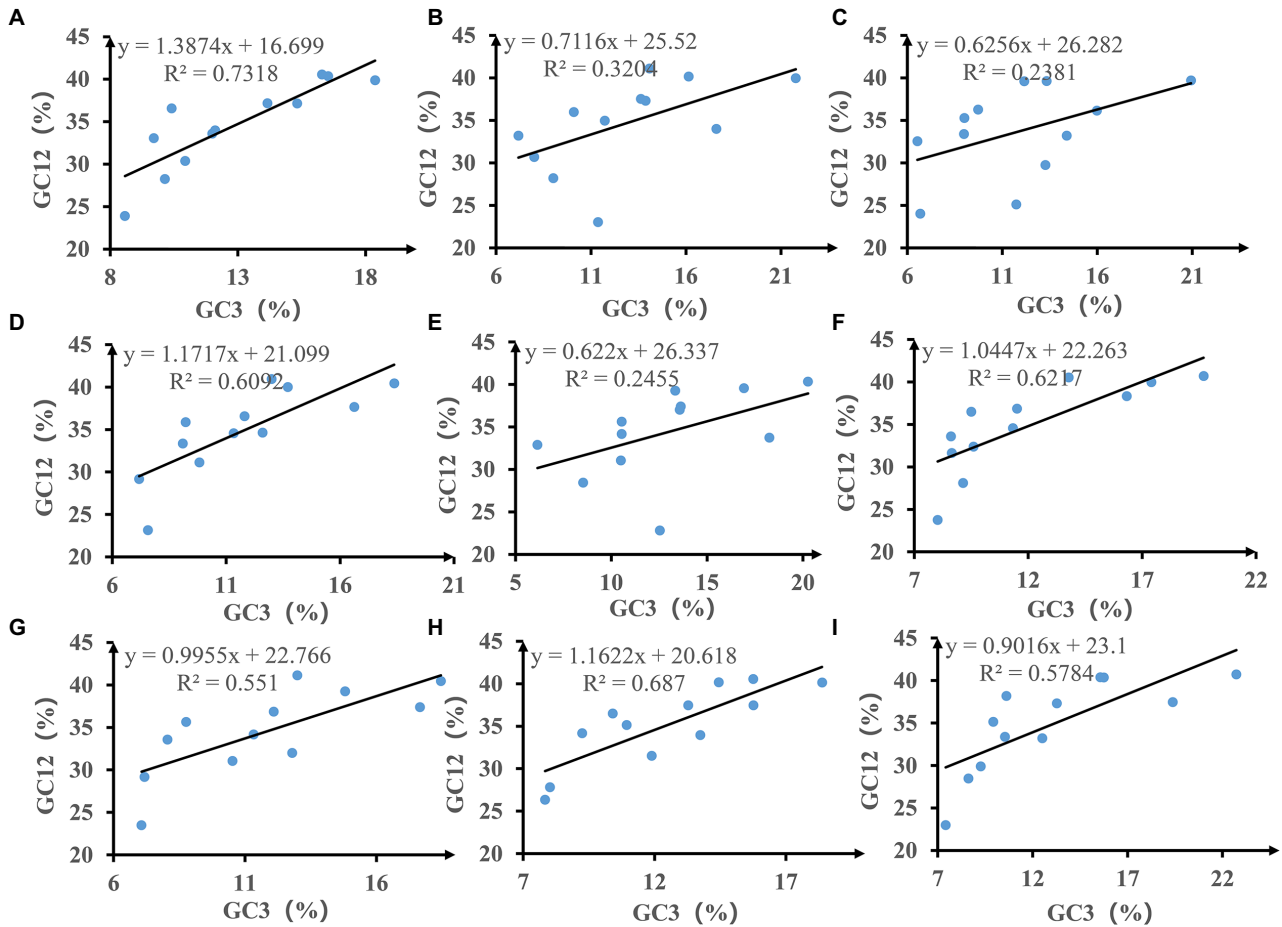
Neutrality plot analysis of GC12 and the third codon position (GC3) for the entire coding DNA sequence of nine *Amanita* species. **(A)**, *A. basii*; **(B)**, *A. bisporigera*; **(C)**, *A. brunnescens*; **(D)**, *A. muscaria*; **(E)**, *A. phalloides*; **(F)**, *A. pseudoporphyria*; **(G)**, *A. sinensis*; **(H)**, *A. inopinata*; and **(I)**, *A. thiersii*.

We calculated the relationships between GC12 and GC3 based on neutrality plot analysis (Figure 3). The GC12 content varied from 22.81 to 41.14%, and the GC3 content varied from 6.14 to 22.73%. The slopes of the regression lines (regression coefficient) ranged from 0.622 to 1.387, indicating that the content of GC12 and GC3 in mitochondrial codons of *Amanita* is weak correlated. In addition, the R^2^ value of the standard curve ranged from 0.2381 to 0.7318. There is no significant correlation between GC12 value and GC3 value (*p* > 0.05), which indicated that natural selection may play an important role in driving the evolution of mitochondrial PCGs in *Amanita*.

#### The effect of ENC on codon bias

The average ENC value of all 12 core PCGs detected ranged from 29.16 to 32.12, with an average ENC value of 30.81 (Figure 1A). The ENC value of all core PCGs was less than 35, indicating that these genes had a strong codon usage preference. In addition, in terms of species, the ENC values of nine *Amanita* species ranged from 30.28 to 31.68, which also indicated that the mitochondrial gene of *Amanita* has a strong preference for codon usage.

**Figure 4:**
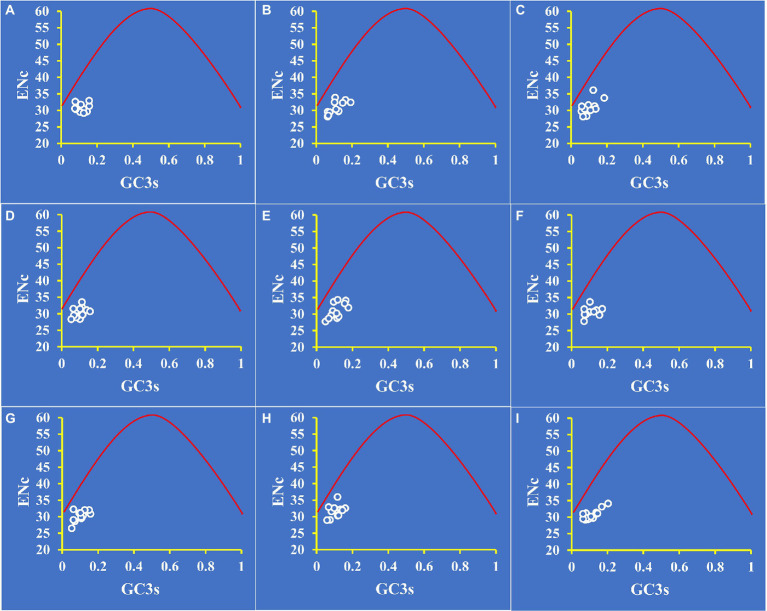
ENC-GC3 plot analysis of 12 core PCGs in nine *Amanita* species. The red solid line represents the expected curve when codon usage bias is affected only by mutation pressure. **(A)**, *A. basii*; **(B)**, *A. bisporigera*; **(C)**, *A. brunnescens*; **(D)**, *A. muscaria*; **(E)**, *A. phalloides*; **(F)**, *A. pseudoporphyria*; **(G)**, *A. sinensis*; **(H)**, *A. inopinata*; and **(I)**, *A. thiersii*.

To distinguish the influence of GC3s on codon bias of *Amanita*, we constructed an ENC plot. Figure 4 shows that all *Amanita* genes were below the expected ENC-plot curve. According to the results, mutational pressure is not the only factor that shapes codon bias; other factors, such as natural selection, also play a key role in codon bias formation.

In addition, we calculated ENC_Ratio_ for mitochondrial core genes in *Amanita* to determine the difference between observed and expected ENC values. As shown in Supplementary Figure S1, the average ENC_Ratio_ values for all core PCGs ranged from 17.70 to 20.65%, indicating that the expected values were greater than the actual values. The GC3s values affected the expected ENC value calculated based on the calculation formula, which further demonstrates that GC3s plays an important role in the formation of codon bias. In conclusion, the results prove that other factors, such as natural selection, determine the formation of *Amanita* codon bias.

#### The preference of third codon

**Figure 5:**
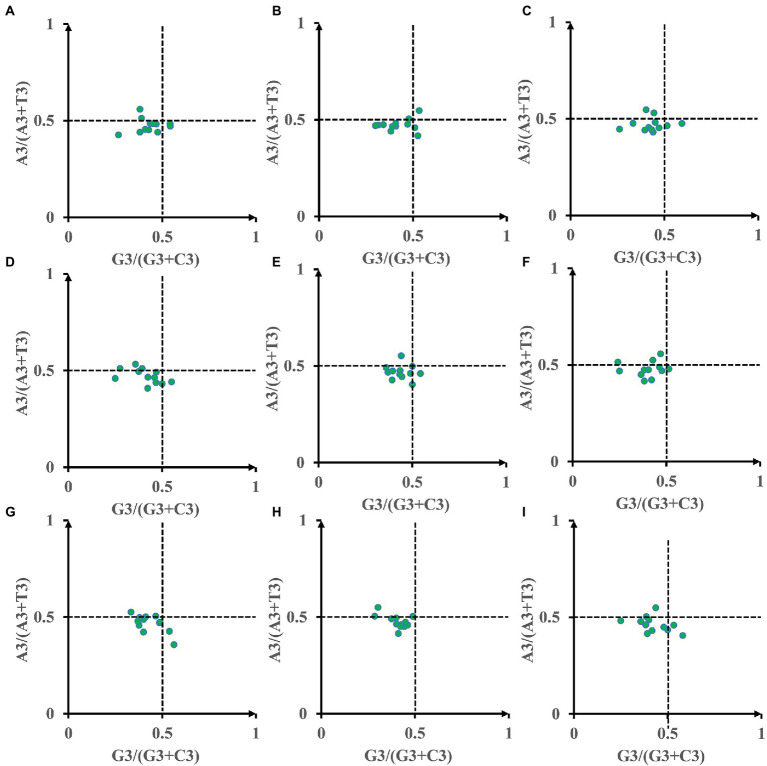
Parity Rule 2 (PR2) plot analysis of 12 core PCGs in 9 *Amanita* species. **(A)**, *A. basii*; **(B)**, *A. bisporigera*; **(C)**, *A. brunnescens*; **(D)**, *A. muscaria*; **(E)**, *A. phalloides*; **(F)**, *A. pseudoporphyria*; **(G)**, *A. sinensis*; **(H)**, *A. inopinata*; and **(I)**, *A. thiersii*.

To determine if *Amanita* mitochondrial genes have biases, we further performed a Parity Rule 2 (PR2) plot analysis (Figure 5). Both axes were centered on 0.5 to divide the plot into four quadrants. Most of the dots were found to be distributed in the third quadrant of the nine *Amanita* species, indicating that the third base of the mitochondrial codon of *Amanita* has a strong preference, preferring T to A and C to G. In addition, eight of the nine *Amanita* species are not distributed in the first quadrant (preferring A to T and G to C), with *A. bisporigera* being the exception (Figure 5B). The results showed that other factors, such as natural selection, play an important role in the process of codon bias in *Amanita* species. The third base of the mitochondrial codon of *Amanita* has a strong preference, which preferred T to A and C to G.

### Correspondence analysis

**Figure 6:**
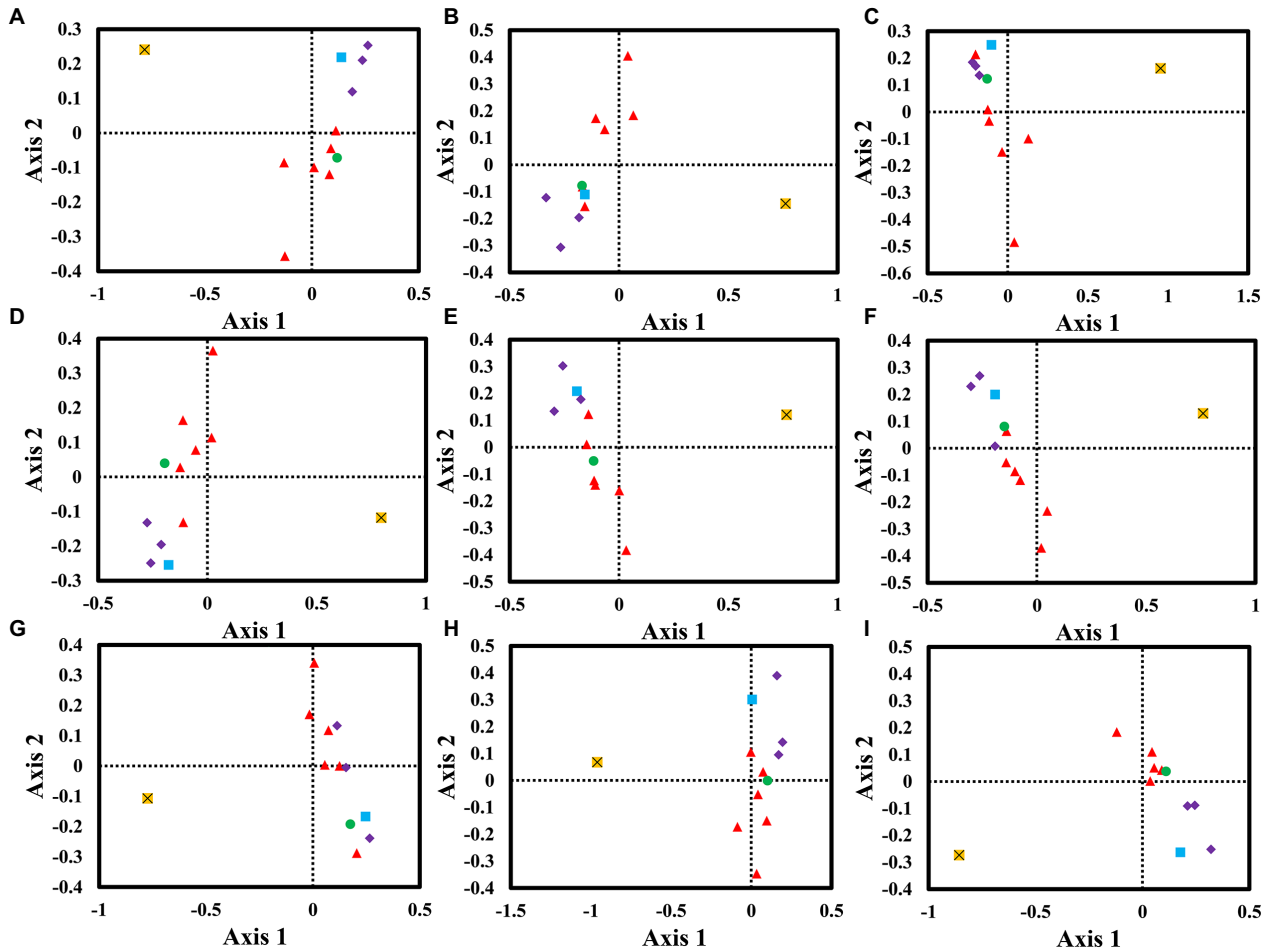
Correspondence analysis (COA) based on the relative synonymous codon usage (RSCU) values of 12 mitochondrial genes from nine *Amanita* species. Purple represents the *cox* gene, red represents the *nad* gene, green represents the *atp6* gene, blue represents the *cob* gene, and yellow represents the *rps3* gene. **(A)**, *A. basii*; **(B)**, *A. bisporigera*; **(C)**, *A. brunnescens*; **(D)**, *A. muscaria*; **(E)**, *A. phalloides*; **(F)**, *A. pseudoporphyria*; **(G)**, *A. sinensis*; **(H)**, *A. inopinata*; and **(I)**, *A. thiersii*.

We performed a correspondence analysis based on the RSCU values of mitochondrial genes from nine *Amanita* species (Figure 6). Axis 1, Axis 2, Axis 3, and Axis 4 are the main contributors to variance, with average contribution rates of 38.61, 16.45, 9.89, and 7.90%, respectively. Among them, Axis 1 is considered to be the largest contributor to variance. We further analyzed the correlation between Axis 1 and GC, GC3s, ENC, CAI, CBI, and FOP. Among the nine *Amanita* species, Axis 1 was found to be significantly correlated with CAI, CBI, FOP, GC, and GC3s (*p* < 0.05), indicating that multiple factors jointly affected the base bias of *Amanita*. In addition, we found that the *rps3* gene and other core PCGs showed large variation, indicating the differentiation of synonymous codon usage of core PCGs.

### Optimal codon determination of *Amanita* mitogenomes

**Figure 7:**
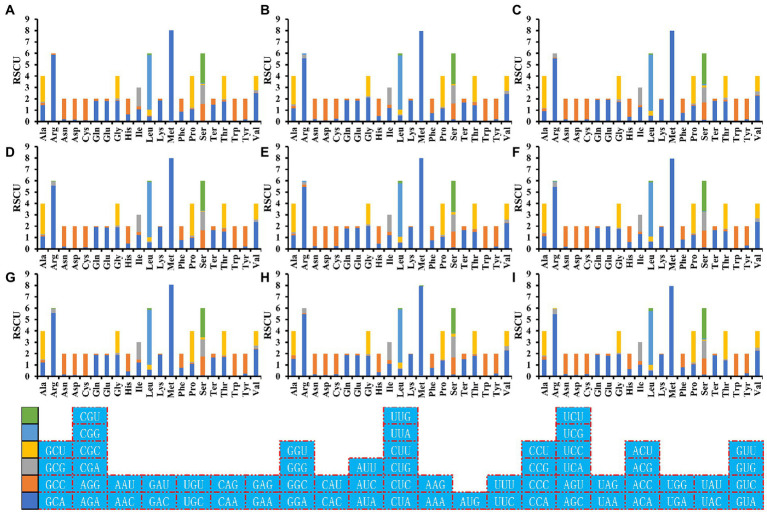
Relative synonymous codon usage (RSCU) analysis of 12 mitochondrial genes from nine *Amanita* species. **(A)**, *A. basii*; **(B)**, *A. bisporigera*; **(C)**, *A. brunnescens*; **(D)**, *A. muscaria*; **(E)**, *A. phalloides*; **(F)**, *A. pseudoporphyria*; **(G)**, *A. sinensis*; **(H)**, *A. inopinata*; and **(I)**, *A. thiersii*.

**Figure 8:**
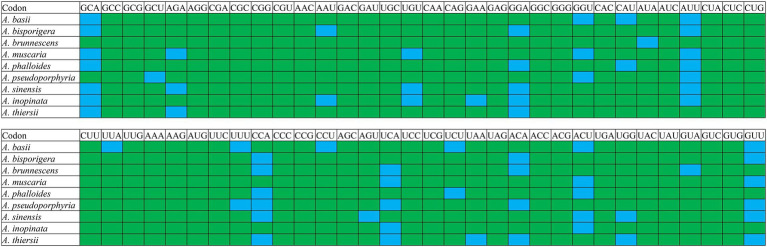
Optimal codons of nine *Amanita* species (ΔRSCU > 0.08 and RSCU > 1), which are marked in blue.

RSCU analysis found that seven of the nine *Amanita* species contained 29 high-frequency codons, including *A. basii*, *A. bisporigera*, *A. phalloides*, *A. pseudoporphyria*, *A. sinensis*, *A. inopinata*, and *A. thiersii* (Figure 7). In addition, two *Amanita* species, *A. brunnescens*, and *A. muscaria*, both contain 28 high-frequency codons because of their RSCU values >1. GCA and CCA are used at a high frequency in other *Amanita* species but they are used at a low frequency in *A. brunnescens* and *A. muscaria*. Among the 29 frequently used codons of *Amanita*, 14 end with T, 13 end with A, and only 2 end with G, indicating that *Amanita* mitochondrial codons tend to end with A/T. In addition, we detected 21, 19, 26, 17, 29, 18, 22, 20, and 26 highly expressed codons (ΔRSCU > 0.08) in nine *Amanita* species: *A. basii*, *A. bisporigera*, *A. brunnescens*, *A. muscaria*, *A. phalloides*, *A. pseudoporphyria*, *A. sinensis*, *A. inopinata*, and *A. thiersii*, respectively. The comparative analysis showed that 10, 7, 5, 8, 7, 8, 10, 8, and 9 optimal codons (ΔRSCU > 0.08 and RSCU > 1) were identified in *A. basii*, *A. bisporigera*, *A. brunnescens*, *A. muscaria*, *A. phalloides*, *A. pseudoporphyria*, *A. sinensis*, *A. inopinata*, and *A. thiersii*, respectively (Figure 8). Most of these optimal codons end with A/T, with the exception of UGG. Among the optimal codons, GCA and AUU are the most widely used, being the optimal codons of 7 *Amanita* species, followed by CCA and GUU, which are used as the optimal codons of 6 *Amanita* species. In addition, GCU, GAA, AUA, UUA, CCU, AGU, UAA, and GUA are each only used as the optimal codons of one species of *Amanita*.

### Phylogenetic analysis

**Figure 9:**
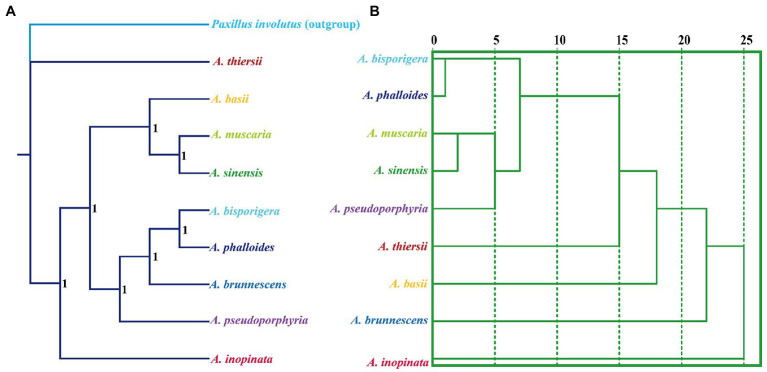
Relationship inference of different *Amanita* species based on the Bayesian inference (BI) **(A)** method and relative synonymous codon usage (RSCU) hierarchical clustering **(B)**. The Bayesian posterior probabilities (BPP) value is displayed on the nodes of the clades.

We used the Bayesian inference (BI) method to construct phylogenetic trees of nine *Amanita* species based on the combined mitochondrial gene set (Figure 9). The results showed that the two saprophytic *Amanita* species, *A. thiersii* and *A. inopinata*, were separated from the *Amanita* population earlier. The symbiotic *Amanita* species can be divided into two main evolutionary clades, where *A. basii*, *A. muscaria,* and *A. sinensis* are one evolutionary clade, while *A. bisporigera*, *A. phalloides*, *A. brunnescens,* and *A. pseudoporphyria* are the other (Figure 9A). Compared with the phylogenetic relationship based on sequences, the species relationship inferred based on RSCU has some differences, such as the phylogenetic status of *A. pseudoporphyria*, *A. thiersii*, *A. basii*, and *A. brunnescens* (Figure 9B). However, the RSCU-based species relationship status also clearly shows the close relationship between *A. bisporigera* and *A. phalloides*, as well as that between *A. muscaria* and *A. sinensis*.

## Discussion

With the development of sequencing technology, researchers can obtain the genetic sequences of various species and types of genomes, including the nuclear genome, chloroplast genome, and mitochondrial genome (Chakraborty et al., 2020; Bao et al., 2022; Tu et al., 2022; Bao et al., 2023). In the process of analyzing the genetic information of species, researchers have found that the probability of synonymous codons of different species being used is not always equal. Some synonymous codons are used more frequently, while some codons are used less frequently (Liu et al., 2020). Codon use bias is mainly affected by many factors, including gene base composition, gene length, gene expression level, tRNA abundance, amino acid hydrophobicity, aromaticity, mutation, and selection, among which mutation and selection are the most important factors affecting codon use bias (Trotta, 2013; Chen et al., 2014). Analyzing the codon bias characteristics and variations of different species is helpful to understanding the genetic structure and evolution trend of species (Yang et al., 2021; Yu et al., 2021). Optimal codon analysis can also provide reference for the genetic expression of host genes. However, to date, the characteristics of codon usage for important organelle genomes of higher fungi have not been fully understood.

The mitochondrial genome is called the “second genome” of eukaryotes. In this study, we found that the length and base composition of mitochondrial core PCGs of different *Amanita* species showed large variations, indicating the differentiation of *Amanita* mitochondrial genes. The difference in synonymous codons is mainly reflected in the difference in the third codon. In this study, we found that all core PCGs of *Amanita* species tend to end with A/T, which is consistent with the rule of mitochondrial codon usage in many eukaryotes (Mazumder et al., 2021; Montana-Lozano et al., 2023). Most high-frequency codons parsed by RSCU also end with A/T, which further proves the tendency of using the third codon of *Amanita*. Different species and genes also show differences in base usage. In addition, we also detected the correlation between codon base composition and CAI, CBI, and FOP, indicating the influence of base composition on codon bias. An ENC value lower than 35 indicates a strong codon preference (Prabha et al., 2017; Pepe and Keersmaecker, 2020). In this study, we found that the average ENC of mitochondrial core PCGs of *Amanita* is 30.81, which demonstrates the strong codon preference of mitochondrial core PCGs of *Amanita*. In addition, the expected ENC value was significantly different from the actual ENC value (17.70–20.65%). The neutrality plot analysis and PR2-Bias plot analysis further demonstrated that natural selection plays an important role in *Amanita* codon bias. The results showed that despite some differences in codon usage indicators among different *Amanita* species, their common point was that mitochondrial PCGs were affected by strong natural selection, which was consistent with the results observed in the mitochondrial genomes of other species.

Mitochondria are believed to have been obtained from alphaproteobacteria by the ancestors of eukaryotes through endosymbiosis (Lang et al., 1999). During the long-term evolution of eukaryotes, most mitochondrial genes have been transferred to the nuclear genome (Adams and Palmer, 2003). At present, most eukaryotes only retain some core PCGs, some tRNA genes and rRNA genes shared by most eukaryotes (Bullerwell et al., 2000; Costa et al., 2012). The mitochondrial genome is considered a powerful tool for inferring the phylogenetic relationships of species because of its advantages (Li et al., 2015, 2022; Johri et al., 2019), including single-parent inheritance, containing many available molecular markers, independent of the origin of the nuclear genome, unique evolution rate, etc. In this study, we analyzed the genetic relationship of different *Amanita* species based on the combined mitochondrial gene set, and each evolutionary clade had a high support rate, indicating the reliability of analyzing fungal phylogenetic relationships based on mitochondrial genetic sequences. In addition, based on the RSCU values of different species, we constructed the relationship between different *Amanita* and found that the relationship between different species based on RSCU was different from the sequence-based relationships. However, the genetic relationship between some species was correctly interpreted according to the RSCU value, which was consistent with other studies (Crane et al., 2021; Gupta et al., 2021). The results show that the phylogenetic results based on RSCU can be an important supplement to the phylogenetic results based on sequence. In conclusion, this study analyzed the base bias characteristics of different *Amanita* species and core mitochondrial PCGs and increased the understanding of codon usage characteristics and genetic evolution of this higher fungal group.

## Data availability statement

The original contributions presented in the study are included in the article/Supplementary material, further inquiries can be directed to the corresponding authors.

## Author contributions

QL: conceived and designed experiments and wrote and reviewed the paper. YL, AS, WX, ZX, JH, and XC: analyzed the data. LZ and LP: project management. All authors contributed to the article and approved the submitted version.

## Conflict of interest

The authors declare that the research was conducted in the absence of any commercial or financial relationships that could be construed as a potential conflict of interest.

## Publisher’s note

All claims expressed in this article are solely those of the authors and do not necessarily represent those of their affiliated organizations, or those of the publisher, the editors and the reviewers. Any product that may be evaluated in this article, or claim that may be made by its manufacturer, is not guaranteed or endorsed by the publisher.
