# Supplementary figures and images for: Analysis of synonymous codon usage patterns in mitochondrial genomes of nine Amanita species
Source: Front Microbiol. 2023 Mar 8;14:1134228. doi: 10.3389/fmicb.2023.1134228 (PMC10030801; doi:10.3389/fmicb.2023.1134228)

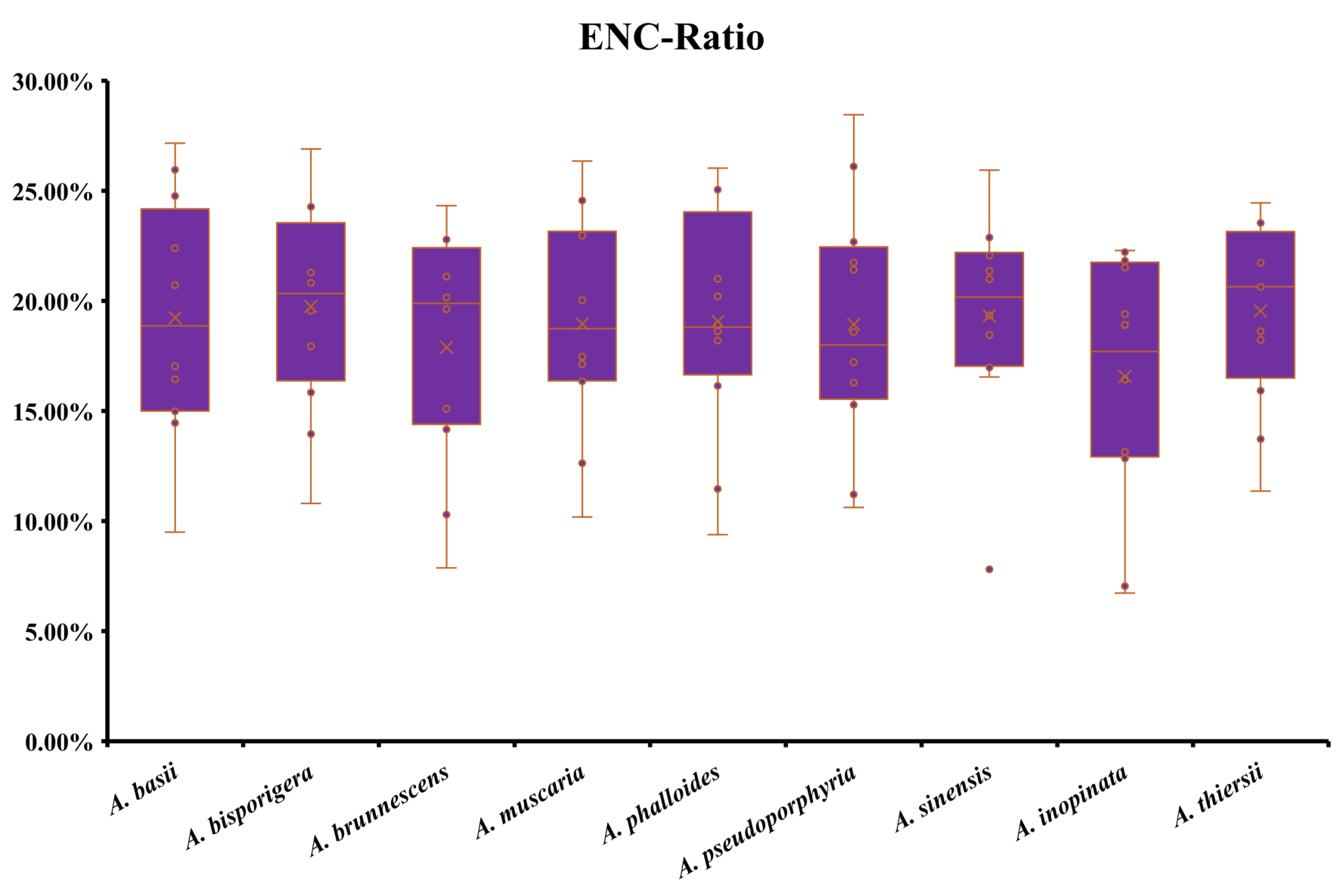

Supplement: Supplementary file 1 [file Figure_S1.TIF]
